# The Impact of Trauma‐Informed Care on Patient Engagement, Experience and Barriers to Care: A Qualitative Study: Empirical Research Qualitative

**DOI:** 10.1002/nop2.70331

**Published:** 2025-09-25

**Authors:** Victoria N. Roberts, Alison M. Hutchinson, Renee L. Fiolet

**Affiliations:** ^1^ Youth and Family, Priority Populations, Monash Health Community, Monash Health Dandenong Victoria Australia; ^2^ School of Nursing and Midwifery, Centre for Quality and Patient Safety Research, Institute for Health Transformation Deakin University Geelong Victoria Australia; ^3^ Barwon Health Geelong Victoria Australia; ^4^ Faculty of Health Sciences University of Southern Denmark Odense Denmark; ^5^ Department of General Practice and Primary Care University of Melbourne Parkville Victoria Australia

**Keywords:** domestic violence, health care, perceptions, sexual assault, trauma, trauma‐informed care

## Abstract

**Aims:**

To explore patient experiences of trauma‐informed care in general health services delivered by community health nurses to individuals impacted by sexual assault or domestic violence, and the impact this care had on service engagement, overall patient experiences and barriers to care.

**Design:**

A naturalistic inquiry approach was adopted.

**Methods:**

Individual semi‐structured interviews were conducted with six adults impacted by sexual assault or family violence attending a multi‐agency metropolitan health service. Data were inductively analysed using reflexive thematic analysis.

**Results:**

The significance of feeling safe and heard was interwoven across three main themes: Enhancing engagement with health care; Sharing the load: the experience of trauma‐informed nursing care; and The nurse's role in breaking down health system barriers and highlighting the value and impact of nurses implementing trauma‐informed care.

**Conclusions:**

Patient experiences supported the need for and provided examples of operationalising trauma‐informed care in health care. Study findings suggest incorporation of trauma‐informed care into mainstream health service provision and training is recommended. Future research should expand understanding of the process and outcomes of implementing trauma‐informed care.

**Impact:**

Trauma‐informed care can prevent re‐traumatisation and disrupt trauma impacts, but implementation in the health sector is poorly understood. This study demonstrates how trauma‐informed care can be translated to general health care settings to improve accessibility to care, enhance engagement and generate positive patient experiences.

**Reporting Method:**

Aligns with the EQUATOR guidelines and COREQ reporting method.

**Patient or Public Contribution:**

Six patients were involved as participants in the study; findings were shared with these patients before being included in the manuscript.

## 1 Introduction

Health care experiences can cause distress and re‐traumatise individuals impacted by trauma, leading to barriers to care, repeat presentations, entrenched symptoms, further harm and increased health expenditure (Jackson et al. 2015; Reeves 2015). Recognising and responding to the impacts of trauma are key to reducing health inequities and facilitating accessible, inclusive and sensitive health care (Fiolet et al. 2021). Recommendations to implement trauma‐informed care (TIC) in health care services emerged from the Royal Commission into Family Violence (State of Victoria 2016) and the Royal Commission into Aged Care Quality and Safety (Commonwealth of Australia 2021). Hegarty et al. (2022, 2019) suggest a whole‐of‐system approach to improve patient outcomes led by health policy, involving an integrated health response and training recommendations, but note the absence of implementation evidence. While health care workers are strategically positioned to reduce the impacts of trauma and support effective and timely treatment, critical research gaps regarding TIC implementation and efficacy to guide health care workers persist (Hegarty et al. 2019; Reeves 2015).

## 2 Background

Trauma is perceived by the individual as a life‐threatening, physically or emotionally harmful event or set of circumstances with lasting adverse effects on functioning, physical, mental, social, emotional, or spiritual wellbeing (Substance Abuse and Mental Health Services Administration 2014, 7). Phoenix Australia (2023), the National Centre of Excellence in Posttraumatic Mental Health, describes sources of trauma as overwhelming and disturbing events characterised by an extreme sense of powerlessness, a disruption of beliefs and expectations, and shattering of basic assumptions with the potential for long‐lasting impacts (Blehm 2024; Janoff‐Bulman 1992).

In Australia, one in four women and one in eight men have experienced violence from an intimate partner or family member, and one in five women and one in 16 men have experienced sexual violence (Australian Bureau of Statistics 2023). One in six women and one in nine men have experienced child sexual abuse before the age of 15, and there is an equal prevalence of people witnessing family violence (Australian Bureau of Statistics 2023). Australian women with a history of sexual violence have higher rates of two or more diseases, including diabetes, reproductive health conditions, asthma and heart disease (Townsend et al. 2022). Epigenetics provides insights on how trauma impacts health as communication between the brain and body occurs through hormonal and neural mediators (Hailes et al. 2019; McEwen et al. 2015). Trauma may result from various experiences, including family and sexual violence, and cause neurobiological changes (Kezelman and Stavropoulos 2018). Unresolved trauma causes disassociation, hypo‐ and hyperarousal, and impacts memory and cognitive processing (Kezelman and Stavropoulos 2018). The prevalence of poor health outcomes associated with trauma is a significant public health issue with substantial costs to society and individuals (Kezelman et al. 2015; Magruder et al. 2017).

Trauma‐informed care (TIC) is a well‐recognised framework in therapeutic trauma services which the Substance Abuse and Mental Health Services Administration (SAMHSA 2014) describes using six core principles (Table 1).

**TABLE 1:** Substance Abuse and Mental Health Services Administration (2014) six core TIC principles.

Six core TIC principles	
1	Safety
2	Trustworthiness and transparency
3	Peer support
4	Collaboration and mutuality
5	Empowerment, voice and choice
6	Cultural, historical and gender issues

Non‐TIC services can result in pathologising, medicalised and patronising experiences (De Boer et al. 2022). Where treatment‐seeking people with complex traumas experience barriers, such as feeling judged, labelled as mad, bad or disempowered, TIC through advocacy can support access with empowerment to establish safety (De Boer et al. 2022). Goldstein et al. (2024) suggest a TIC approach in health care is critical to promoting safety and preventing traumatisation and highlight the need for the evaluation of TIC approaches. The health impacts of trauma and potential to reduce the distress associated with health interventions, provide a strong rationale for exploring TIC in a health care setting. Implementation evidence will inform whether TIC can prevent unnecessary distress and interrupt the trajectory of trauma associated harm. TIC frameworks suggest implementation requires a systems response through organisational transformation at all levels (State of Victoria 2023).

Multidisciplinary Centres (MDCs) consist of collocated, collaborative agencies to address barriers to reporting and support people affected by family violence and/or sexual offences. Barriers to reporting include feelings of shame, guilt, embarrassment, not being believed, confidentiality and not wanting family to know (Sable et al. 2006). Community health nurses working in MDCs have embedded TIC in their practice. Adoption of TIC in a health care setting provides a unique opportunity to explore the impact on patient experience, engagement and reducing barriers to health care access. Furthermore, it provides an opportunity to inform future implementation of TIC strategies and research in other health settings.

### 2.1 This Study

#### 2.1.1 Aim

We aimed to explore patient experiences of trauma‐informed care delivered by community health nurses to individuals impacted by sexual assault or domestic violence, the impact this care has on service engagement, barriers to care, and to understand potential transferability across the health service sector.

The question addressed in this research was: *How does TIC impact service engagement, experience and barriers to care?* The study aligns with research recommendations from Hegarty et al. (2022) for targeting critical gaps in the evidence.

## 3 Methods

### 3.1 Design

A naturalistic inquiry approach was used in the design of this qualitative study, using semi‐structured interviews to explore patient perceptions of the operationalisation and impact of TIC. This method was chosen to obtain rich descriptions of individual experiences and develop an in‐depth understanding of the complex and sensitive topic (Jameel et al. 2018).

### 3.2 Study Setting

The research was conducted in a Community Health Nurse (CHN) Program within Multidisciplinary Centres (MDCs) from a metropolitan public health service in Melbourne, Australia (State Government of Victoria, Victoria Police 2025). MDCs co‐locate a range of partner agencies to provide a patient‐centred, integrated and holistic service response in an environment that provides safety, support and access to justice (State of Victoria, Department of Health and Human Services 2019). The CHN role within the MDC is strategically positioned to work with trauma survivors of family violence and sexual assault using trauma‐informed care. MDC nurses have consolidated TIC knowledge and implement the core values by applying them to processes, practice and being agile in anticipating needs and providing individualised TIC. In this setting, the CHNs implement TIC while using a social model of health to enable health needs identification, care planning, referral to appropriate service providers, education, awareness‐raising and through the development of local networks of care (Department of Health and Human Services, 2019).

### 3.3 Recruitment

Recruitment facilitated by MDC nurses involved the identification of potential participants from a sample of 192 patients who met the eligibility criteria (Table 2).

**TABLE 2:** Eligibility criteria.

Eligibility criteria	
1	Adult trauma survivors of sexual assault and/or family violence who had engaged with the program in 2020 or 2021
2	Had a health care plan
3	Able to read and understand English
4	Not currently in crisis

The MDC nurses were instructed to only identify eligible participants who had completed their full episode of care, rather than those who were still receiving some sort of support or follow‐up care, to reduce the ethical risk involved with further exacerbating distress. Eleven eligible patients were identified and contacted by the researcher to discuss the opportunity further, provide an information and consent form and the opportunity to ask questions. Six patients provided written or verbal consent to participate before interviews were conducted. Their ages ranged from 19 to 72; the mean age was 38. All interviews were conducted by phone and data collected at the workplace and were informed by an interview guide (Table 3). The interviews were undertaken by the first author, an experienced registered nurse and master's prepared social worker with extensive experience in this field who was mentored by two PHD prepared nurse researchers. The Audio recordings were transcribed verbatim by an external professional transcription service with training and expertise in handling sensitive material. No additional notes were taken, and the time taken was sufficient to complete the interview.

**TABLE 3:** Interview guide.

Victim‐survivor interview guide	
1	How did you find out about the CHN service?
2	What motivated you to receive support from the MDC community health nurse?
3	What made it easy for you to work with the MDC nurse?
4	What other health services have you accessed prior to the MDC nursing service?
5	Can you tell us about anything that may have made accessing health services difficult before using the MDC nursing service?
6	Can you describe your experience with the MDC nursing service?
7	How would you compare the MDC nursing service to other experiences of health services?
8	Is there something that the MDC nursing service provided that is different to your other experiences of health?
9	When working with the MDC nurse, what have you found valuable?
10	When working with the MDC nurse, what did you not find valuable?
11	What could be done differently in the service?
12	Is there anything else you would like to tell me about the MDC nursing service?

### 3.4 Data Analysis and Rigour

Braun and Clarke's (2006) six‐step thematic analysis approach guided the inductive data analysis. The principal researcher repeatedly read the transcripts and undertook the initial data‐driven manual coding. The research team independently read the transcripts, reviewed and revised the coding framework until consensus on the codes was achieved. Codes were grouped into categories by the research team; the themes were then collaboratively developed with several iterations and revisions until a final consensus was achieved. The data were managed using the NVivo software program. A two‐page participant infographic summary of findings was disseminated to all participants who consented to receive the findings.

The researchers who performed the analysis were all trained registered nurses engaged in fields of nursing practice. During this study, two researchers held positions as nurse researchers and the principal researcher held the role of state MDC nurse coordinator responsible for programme strategy, education, research, nursing support, and oversight but not providing direct patient care. In addition, the collective insights of the researchers included practice experience, lived experience of sexual assault, and subject matter expertise of sexual violence and domestic and family violence, which allowed for an in‐depth understanding of the data.

### 3.5 Ethical Considerations

Data from this study was collected after receiving approval from the REDACTED Health Human Research Ethics Committee (approval number HREC/77451/MonH‐2021‐281029(v2)). Informed consent from research participants was obtained before participation and confirmed verbally at the commencement of the interview, as evidenced in the transcripts. Purposeful sampling and use of eligibility criteria to recruit interview participants recognised the vulnerability of victims/survivors of sexual assault and family violence to support participant safety and the recruitment of information‐rich cases. Limited demographic data were collected to acknowledge participant sensitivities to disclosing personal information and to protect participant privacy due to the sensitive nature of the research. The interviewer was a trauma‐trained, experienced researcher who has worked with victims/survivors of family violence. To respond appropriately to participants experiencing emotional discomfort, a distress protocol developed by the researchers for this specific study was available for the researcher to use if required and outlined the appropriate steps for managing discomfort.

## 4 Results

Six individuals participated in this study; five of these were between 18 and 65 years of age, with one individual above 65. Of these, one participant was male, and the remaining were female. Participant experiences of TIC in a health service setting suggest that patients felt safety and trustworthiness, which were described across three main themes: *Enhancing engagement with health care; Sharing the load—the experience of trauma‐informed nursing; The nurse's role in breaking down health system barriers*. Each main theme represented aspects of the operationalisation of TIC in a health service. An in‐depth understanding of factors influencing engagement, experience, and barriers to access is evidenced in seven subthemes across the three main themes. The study methods and themes were shared through an infographic and figure shared with the six participants for feedback (see Data S1).

**FIGURE 1:**
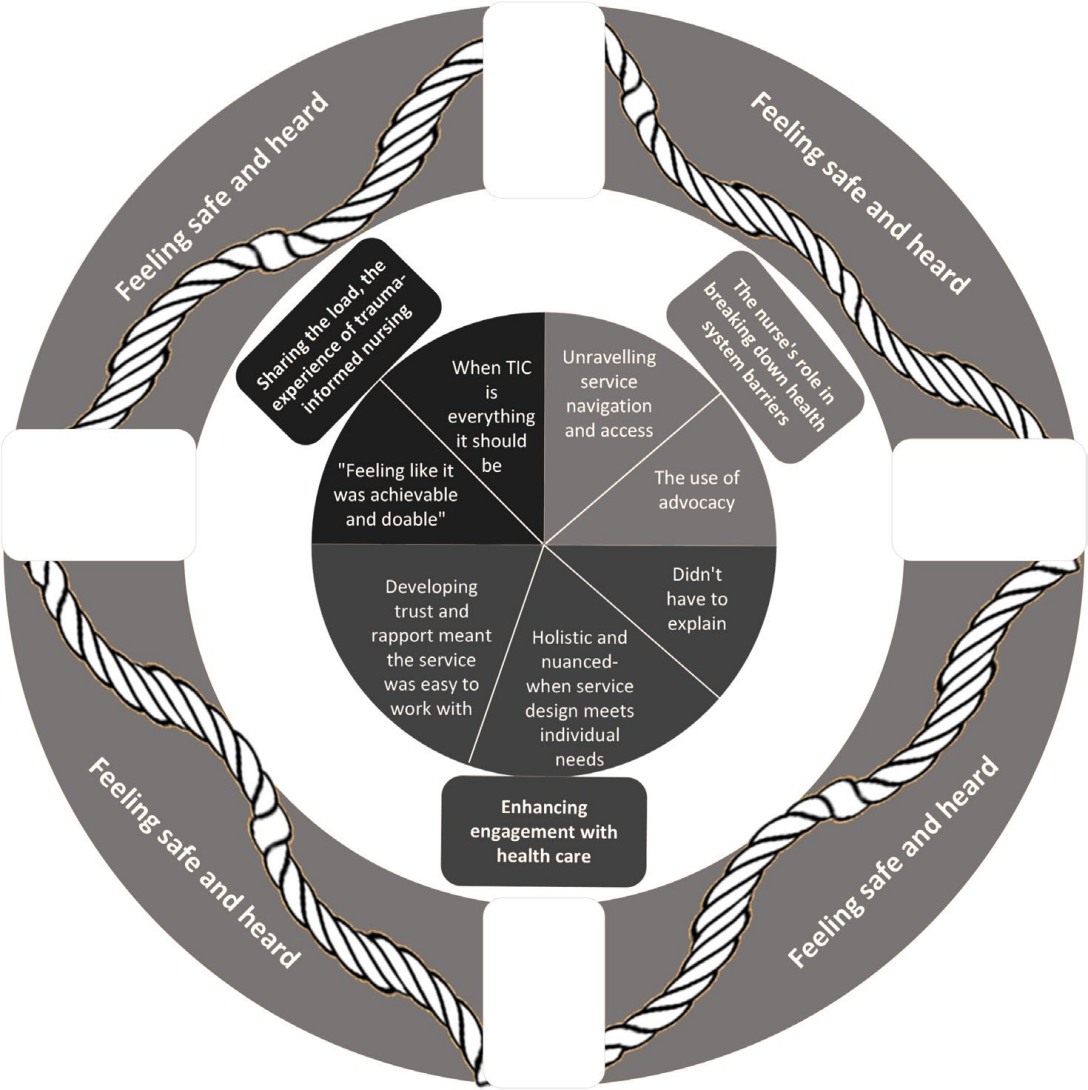
Themes and subthemes.

The themes and subthemes derived from patient experiences of TIC in health are illustrated in Figure 1, with the outer circle showing that the main message is that participants.

### 4.1 Enhancing Engagement With Health Care

The theme, *enhancing engagement with health care*, reflects participants' engagement with the MDC community nursing service and its pivotal role in contributing to improving health outcomes. Engagement was perceived to be an important part of participants' care, and they felt more engaged when the nurse provided holistic care, recognised their needs, and developed relationships that fostered trust. Engaging with services was influenced by the service providers' ability to understand the impacts of trauma and needs. This knowledge informed an ongoing assessment and response to the individual needs, which facilitated engagement of the patient. Knowledge and responsiveness contributed to the development of trust, which was an essential component identified by participants from their experiences. The three subthemes were: *Holistic and nuanced—when service design meets individual needs; Didn't have to explain*; and *Developing trust and rapport meant the service was easy to work with*.

#### 4.1.1 Holistic and Nuanced—When Service Design Meets Individual Needs

The subtheme, *Holistic and nuanced—when service design meets individual needs*, reflects participants' perceptions of factors that enhanced their relationship with health services. Participants felt empowered when engaging with a *s*ervice that was ‘more flexible and able to be nuanced depending on what I needed’ (P2) and emphasised the importance of an individualised response. They also highlighted aspects of the service design that they deemed important, including service integration, co‐location, personalised intervention, where the providers have time, are aware of, and equipped to respond to trauma:

It was very personal. It was almost like the service had been designed personally for me, kind of. It wasn't – because she was wonderful, for the – it was almost like she was a friend that was kind of guiding me through this period of my life. (P. 6)

Participants expressed high regard for being able to choose where health care could be delivered across a range of environments, demonstrating value for empowering actions used by nurses. Participants perceived that the nurse supported them across various needs, from advocacy within appointments to meeting in a park for safety purposes. In describing the design of health care interventions, some participants described feeling empowered, safe, supported, and informed when receiving care based on the provision of choice, ability to accommodate their needs, communicate and provide information effectively.

Vulnerability underpinned individual needs:

It's layers of vulnerability added on top of vulnerability, on top of vulnerability, so the way that we engage with people in that space has to be extra aware… when working with vulnerable people in their very fragile state. (P. 2)

All participants discussed the negative impact of trauma, which included symptoms such as emotional distress, agoraphobia, physical health problems, eating disorders, suicidality, mental health conditions, homelessness and health conditions requiring hospital admission.

Participant five's insights help illustrate: ‘*…* if another person, another party is there to listen (to the) whole scenario in a calm manner, that gives you a lot more relaxation in your life’ (P5).

Sentiments which were echoed by other participants that feeling heard resulted from having a direct line of communication with the nurse who listened, was supportive, demonstrated respect, and was available and accessible. Some participants described experiences with the nurse where the nurse used a curious enquiry whilst exploring their priorities and their perspective on the supports they would need:

[the nurse was] very curious just to find out what kinds of things I needed in terms of support and, now, accessing health services, and what would be the best way to support me in doing that. (P2)

Some participants articulated negative experiences when the service could not meet their individual needs due to the impact of the COVID‐19 pandemic restrictions on MDC nursing operations. These impacts included not having the service during the pandemic, a lack of health information and difficulty navigating and engaging with health care services:

I had a nurse, and that is the one that I met at the park and everything. I was starting to develop a really good rapport and especially straight after the mental breakdown that caused me needing to get into this service, but she got moved to a different district, and then I kind of got lost in the system. (P1)

Participants perceived kindness, genuine concern and feeling welcome through their interactions, influencing their perceptions of safety. Some participants described the value of communication through a holistic health assessment. Participant 3 noted that the nurse looked for cues, including how they looked physically, and regularly assessed their health and physical wellbeing:

…she'd always assess how I looked and ask me, are you okay or something? Ask if I have food to use and all my living conditions; where I live, whom I live with. It was generally about my wellbeing I would say. I felt it was very—a feeling that you feel supported, heard and appreciated the way you are… (P3)

Some participants described the value of being given written information so they did not become overwhelmed, including phone numbers to call, which they would refer to later.

#### 4.1.2 ‘Didn't Have to Explain’

The subtheme, *didn't have to explain*, depicts the importance of health care providers understanding what individuals may experience following traumatic events, and how essential it is to avoid retraumatisation. Receiving care from nurses who understood trauma meant patients did not have to explain their reactions, which could otherwise be misinterpreted. As nurses demonstrated foundational knowledge and understanding of trauma, it removed the participant burden of educating the health care provider:

Because the CHN was trauma‐informed, that she didn't need to be educated on that made my experience feel much more safe and—yeah, safe really. (P2)

‘I was experiencing very debilitating panic, and so that was making it difficult to engage with health’ (P2) is how participant 2 describes the paralysing anxiety and becoming overwhelmed. In this context, participants required support for emotional safety when they were unable to explain what they needed. Similarly, participant 3 adds ‘because by then I really used to get emotional and sometimes it was difficult to explain myself’ (P3). Experiencing emotional safety was perceived to result from health care providers knowing how to support emotional safety through service delivery. The burden of explaining emotional reactions to no obvious precursor was also described ‘So, in the context of filling in a form, there were times that filling in a form would have made me have a full‐scale meltdown’ (P2). In the context of feeling very vulnerable, overwhelmed, positive experiences of the MDC nurse were described: ‘Definitely I felt that throughout. There wasn't any time that I felt that the nurse had abandoned or ditched me throughout the program. She was really good at checking in on how I'm doing’ (P2).

Participants described an environment favouring emotional safety to have the following characteristics: offer support, offer listening, be welcoming, and provide a sense of being taken seriously and validated.

An MDC nurses' knowledge about supporting individuals with past trauma and developing trust surpassed that demonstrated by other health professionals as described by participant 6:

I think that was, you know, just not – well, her knowledge and skill as well, but I guess her own personality, and perhaps because she was working at (…) she obviously had previous experience with people with trauma. Yeah. So, she was quite – I think, pretty specialized in her knowledge of how to deal with past traumas. (P6)

Participants repeatedly referred to the nurses' knowledge, people skills and personal attributes ‘the nurse, herself, was very personable and very warm, very empathetic’ (P2), ‘Yeah, no, it was just about how it was super‐welcoming and like she was really kind’ (P4). ‘It was very, very positive. Not just her nursing skills, but her, basically, general—her knowledge and her people skills were outstanding’ (P6). These skills and qualities were identified as favourable, contributing to reassurance and essential to a positive care experience. Participants shared that the emotional burden of trauma impacted their mental health and ability to function, which negatively impacted their motivation and often resulted in isolation. Participants perceived that the nurses understood trauma impacts, monitored their wellbeing and anticipated needs. All participants, excluding one, described the positive impacts of being understood, keeping them on track, and being supported to understand how trauma impacted their physical health problems and life. This contrasted with participant experiences of other health settings where they found it difficult to have positive experiences and felt dismissed: ‘if the psychologist found out I was referred from a CATT (Crisis Assessment Treatment Team) team, they actually left me’ (P1).

#### 4.1.3 Developing Trust and Rapport Meant the Service Was Easy to Work With

The subtheme, *developing trust and rapport*, *meant the service was easy to work with*, reflects participants' perceptions of what contributed to their engagement with the MDC community health nurse. The apprehension experienced by patients when entering the service is illustrated in this statement: ‘Initially, I was hesitant because I wasn't really trusting because I wasn't sure the reception I'd get’ (P3). Participants described the importance of trust:

Apart from the – her knowledge that helped me physically – my physical difficulties, but I very quickly developed a trust with her that enabled me to ask her to go to the doctor's appointment with me. So, I think that for me – that was the most important part, or the most important thing she offered. (P6)

Participant 6 reflected on a previous negative experience with a psychiatrist where they were not validated, resulting in a breach of trust and their inability to seek support for decades, which led to significant health impacts.

Participant perceptions of developing trust were associated with experiences of always feeling involved in their care, being listened to, validated, taken seriously, where nurses were reliable and consistent, as expressed by one participant, ‘whatever I spoke about was like taken seriously and was listened to and validated’ (P4). Participant 2 stated, ‘…the nurse, herself, was very personable and very warm, very empathetic and very—as far as what my experience was—very willing to support in whatever way was needed’ (P2).

On the other hand, participant 3 reflected on developing trust: ‘Initially, I was hesitant because I wasn't really trusting because I wasn't sure the reception I'd get. I wasn't sure about the support I was going to get as well’, which was relieved: ‘Through her help and her being there and making follow‐up and checking on me, the short time we had together I can say it was very good because so far, I'm okay [because] of that treatment’ (P3). Experiences of safety that supported the development of trust were attributed to communication strategies: ‘I loved her way of communication; the way she guided me in a very calm manner’ (P5). Participant 2 shared that having goal setting on paper helped provide structure and containment, providing clarity about direction and what to expect. Participants described being able to trust what was communicated by the nurse based on previous experience that the nurse delivered on what was promised: ‘the follow‐up with the nurse was very good; if she committed to doing something, she did’ (P2). Most participants spoke of strategies to limit the sharing of sensitive information and protect confidentiality in families and valued knowing that the nurse was concerned for their privacy and actively involved in protecting it. While reflecting on the impact of their ‘mental breakdown’, participant 1 indicated that they successfully developed a good rapport with the nurse. Some participants experienced being introduced to the nurse by someone they trusted, and they perceived that this influenced their ability to trust.

### 4.2 The Nurses' Role in Breaking Down Health System Barriers

The theme, *nurses' role in breaking down health system barriers*, reflects experiences of the nurses' role in supporting access to health services. Experiences of trauma resulting in additional barriers to accessing health care were well described, and the influences of TIC are evidenced through descriptions of overcoming these barriers. TIC influencing barriers is reflected in two sub‐themes: *the use of advocacy* and *unravelling service navigation and access*.

#### 4.2.1 The Use of Advocacy

The theme, *the use of advocacy* reflects participants' unanimous perceptions of nurses' advocacy positively impacting their health care experience. Participants shared that advocacy involved simple initiatives that supported them in overcoming health barriers:

‘One of my big barriers was I didn't have enough confidence to go to the appointment by myself’, and a feeling of safety was associated with knowing ‘I wasn't alone’ (P1). Participants described the need for support through advocacy and the difficulties experienced in identifying a trusted person with whom they could confidently share sensitive information. Perceptions of safety in communication were evident in participants' descriptions of overcoming obstacles and initiating sensitive disclosures:

It would be easier for me to discuss these things with the GP, if there was somebody else there, who was not part of my immediate family, because I haven't told any of them about my past history. (P6)

A common experience described by participants was being unable to think when overcome by emotion, resulting in communication deficits that rendered them unable to articulate or process information. Participant 5 felt the nurse listened to their perspective and was effective in ‘dealing for me’ when they could not think clearly. Advocacy was also described as helpful to make oneself understood, ‘I really used to get emotional and sometimes it was difficult to explain myself’ (P3). Nurse advocacy removed a barrier to engagement with dental care for one participant: ‘she (the nurse) was able to communicate to the practitioner in one brief sentence this is the approach that would be most helpful and why’ (P2). Participant 3 described how the nurse advocated using clinical knowledge regarding symptoms and their outstanding concerns to obtain further investigation and treatment:

When I met the nurse; I still had the symptoms. When I shared with her, she took me to the right – to another doctor again, whom I feel through her systems, I got to be treated well because it was something that as I was told, it was manifesting, and I didn't really know what it was. (P3)

They contrasted this with their experience of ineffective communication when conveying information to the doctor alone. The participant attributed their ability to overcome communication barriers to the nurse's presence and the nurse's ability to convey accurate information as a patient advocate. Participants perceived the MDC nurse as protective against power imbalances when present to advocate in an appointment and felt it diffused the power dynamic held by the other health professionals compared to attending on their own. Some participants gave examples of how the nurse could advocate for their safety with others and negotiate strategies to achieve this. Participant 2 reflected that having the nurse present, bearing witness, increased the likelihood the health practitioner would consider the patient's specific needs and modify their approach; it also assisted with making appointments and advocating for services to accept the government funding as full payment with no out of pocket expenses, also known as ‘bulk billing’.

#### 4.2.2 Unravelling Service Navigation and Access

Participants describe how the MDC CHN service uses nurses' clinical knowledge, sector information, and interventions to support access. As illustrated by participant 1, ‘Without me becoming overwhelmed with the information, she would just break it down and then send me two lists, write a little bit about them, and then it was up to me to decide which one’ (P1). Some participants found the nurse supporting access helped them with their lack of knowledge, motivation, or inability to take the first step. Participants shared observations that the nurse had a good knowledge of health‐related services in the local area, and participant 2 reflected, ‘it empowered me to access further supports that I wouldn't have otherwise known were available or I wouldn't have been able to ask for support or help.’ Participants perceived that acquiring this knowledge empowered them and resulted in access to services that would not have been accessible otherwise, ‘I guess just the lack of knowledge around services would have been a challenge, I guess. Maybe unsure of like even if I did know the services, just like unsure of what the services entailed, I guess.’ (P4).

### 4.3 ‘Sharing the load’: the Experience of Trauma‐Informed Nursing

Participants reported experiences of hope, empowerment, control and capacity, as reflected in the theme branded using participant 2's expression: ‘Sharing the load’: *the experience of trauma‐informed nursing*. When nurses facilitated care that met individual needs to support engagement and access, participants perceived they could share the heavy weight associated with trauma. In understanding the experience of trauma‐informed care, two subthemes were identified: ‘When TIC is everything it should be’ and ‘Feeling like it was achievable and doable’.

#### 4.3.1 When TIC Is Everything It Should Be

Most participants described their encounter with the MDC nurse as positive: ‘when TIC is everything it should be’ (P2). Being able to share the load also represents other participants' perceptions of feeling ‘safe, really safe’ because they were being cared for by ‘a great service and relieved me of so much stress’ (P5). According to Participant 1, expressing that sharing the load ‘made me feel pretty good’. Some participants expressed that having a positive experience was mostly because ‘I felt it was very– a feeling that you feel supported, heard and appreciated the way you are’ (P3). Participant 1 expressed the significance of the nursing providing TIC: ‘I felt like it made a big difference by actually seeing her.’ And described how the nurse assisted them in navigating care: ‘Then also, just practically, she was a local, and so she had a really good knowledge of the local area, the local services. Yeah, that made it obviously a lot easier as well because I had no clue [laughs]’ (P1). Participant 2 reflected that ‘a lot of the support services were, outside of the community health nurse, were very rigid and imposed. This was a lot more flexible and able to be nuanced depending on what I needed.’ Participants also described TIC as a holistic experience, ‘more trauma‐informed for sure’ (P2), where descriptors of care experiences included ‘personalised’ (P6), ‘trustworthy’ (P5), ‘supportive’ (P1) and ‘timely’ (P2).

#### 4.3.2 ‘Feeling Like It is Achievable and Doable’

The subtheme, ‘Feeling like it was achievable and doable’, using the words of Participant 2, captures aspects of perceived service impacts as meeting patient needs at the time. Some participants described the characteristics of the care they found most valuable. Participants described the ease of access to the nurse, and that nurse availability for appointments made it easier to engage with health services. Supporting health care navigation was also considered valuable when the nurse collated and simplified the information, providing them with a couple of service options that matched their needs. Participants suggested that the nurse role was helpful, the nurse ensured they would ‘be able to get the right support in the system’ (P3). Sentiments about nurse influencing motivation ‘I don't think I had any motivation, but she didn't give up on me, which is something I really liked’ (P1). The service design and principles were attributed to making it ‘really easy to work with’ (P4). Participants valued collaboration between the counsellor and nurse, linking service integration with trauma recovery. Reflecting on the competing pandemic priorities, participants identified the importance of clear communication in service delivery and highlighted the increased vulnerability and additional support required during transition periods in and out of services. When the MDC nursing program was limited to a short duration or pandemic‐related abrupt cessation of the service, participants described associated ‘rough’ experiences without the support.

## 5 Discussion

Implementing TIC in a health care setting through a practice framework that addresses knowledge, understanding, safety, relationships, client voice, avoidance of re‐traumatisation, the use of reflection, and a holistic approach that focuses on wellbeing is an emerging priority for health care services (State of Victoria 2023, 26). TIC, using core values, strives to prevent a triggering event or circumstances that bring to memory an earlier trauma, also known as re‐traumatisation (SAMSHA 2017; Goldstein et al. 2024). Whilst there is growing recognition that TIC principles should underpin every interaction, practice, program, and service supporting people across the health and human service system, the literature is devoid of practice examples in generalist health care settings (State of Victoria 2023).

### 5.1 TIC In Health Settings

The community health nursing program in this study forms part of a continuum of care within the MDC that aims to respond to the physical and emotional health and well‐being of patients who have experienced sexual assault or domestic violence by implementing TIC. In contrast, outside the walls of the MDC, most other health care services do not operate from a TIC framework; thus, impacting their ability to be accessible and adequately address trauma‐related needs. The prioritisation of safety found in this study of TIC is key to preventing and addressing stress responses for individuals with traumatic experiences, and implementation findings provide valuable insights to increase understanding of the operationalisation and experiences of TIC in health care (Oberle et al. 2021).

Prioritising safety includes emotional safety, reducing further harm, and overcoming barriers to health care accessibility resulting from mistrust, disengagement, and re‐traumatisation (Chaudhri et al. 2019). TIC has been attributed to influencing cognition, decision‐making, anxiety, mood and behaviour states (McEwen et al. 2015). Establishing safety through TIC is a targeted approach to addressing key sensory inputs perceived as dangers responsible for the stress response (Dowdell and Speck 2022). Consequently, addressing trauma impacts through TIC can positively influence engagement, experience and barriers to accessing health care services, and such findings strengthen the evidence for implementing TIC in health services. Findings from this study are derived from participants' experiences where TIC was perceived to support adaptation in response to traumatic events.

The findings of this study suggest that implementing TIC in health requires both a system and individualised approach consistent with the implementation in mental health services (SAMSHA 2023). Specifically, the delivery of TIC in health requires a foundational understanding of trauma, including identifying risks to safety, emerging stress responses, and responding to an individual's needs. TIC described in this study has emerging themes illustrating concepts of TIC core principles; the TIC framework described by Kezelman and Stavropoulos' (2018) includes safety, trust, choice, collaboration and empowerment. Study findings suggest when nurses implement TIC, it supports successful engagement, positive experiences, and reduces barriers to accessing health services. This is an important finding for ‘never‐served’ persons who avoid health services because they have previously experienced judgement, disempowerment and exclusion, compounding the effects of traumatic stress (Dowdell and Speck 2022).

### 5.2 Implementing TIC in Health

Positive experiences of safety were associated with health service providers who understood the need to be trauma‐informed and invested in service capacity to offer TIC. Furthermore, trust established through TIC reduced the burden on the individual to educate the service provider about their experience of trauma, its impacts and how the service can become safe for them to access. Evidence from this study highlights empowerment and collaboration through TIC by removing barriers to accessing health services through clinician advocacy, skills and clinical knowledge to identify appropriate resources to meet individual needs. Another key finding of this research was the value of safety where participants didn't have to explain their circumstances and defend their feelings or behavioural responses. De Boer et al. (2022) suggest that subtle experiences of acceptance and connection through trust and transparency that can help trauma survivors feel understood, and this is supported in the current study. As established in this study, TIC provides an opportunity to address the impacts of trauma and overcome disrupted human connection resulting from not feeling understood and, consequently, feeling alone, supporting the findings of Wilde (2022). Participants in this study felt supported to engage with health services by not having to explain when they felt overwhelmed, emotionally vulnerable, or could not articulate what they needed, illustrating safety. These findings are supported by existing research affirming the importance of safety through being understood to facilitate engagement and enable individuals to make sense of their symptoms (De Boer et al. 2022).

Emerging evidence of TIC implementation in health care from this study suggests that TIC involves a tailored, individualised approach (SAMHSA 2014; State of Victoria 2023). Participant experiences of enhanced engagement through appropriately tailored interventions derived from the assessment of their individual needs highlight the significance of understanding the individual experience to inform TIC (State of Victoria 2023). Recognising that it is the individual experience of an event which results in trauma that requires developing strategies to address individual needs means that TIC is not a ‘one size fits all’ solution (SAMHSA 2014). Findings suggest though TIC principles endure, effectively implementing *the use of advocacy* supporting empowerment to create an experience of *feeling like it was achievable and doable* through collaboration requires the identification of individual characteristics that direct care. A TIC service with trust and transparency and described as *holistic and nuanced—when service design meets individual needs—demonstrates* the ability to be dynamic and tailored in response to individual needs. The importance of a tailored approach identified in this study is congruent with recommendations from Nation et al. (2022), who postulated that TIC implementation requires an individualised, longitudinal and holistic perspective for each patient. Study participants identified the need for contextualised responses specific to their requirements and informed by their lived experience. Positive experiences of having individual needs met through collaboration and not having to explain their trauma contrast with negative patient experiences of service providers who failed to understand their circumstances, highlighting the need for TIC in health care across all levels (De Boer et al. 2022; SAMHSA 2023).

A key finding of this research was the critical role of trust, consistent with the evidence to establish safety and a therapeutic, trusting relationship to prevent re‐traumatisation and disengagement (Chaudhri et al. 2019). Building a strong therapeutic relationship is critical to engagement and meaningful treatment (De Boer et al. 2022). Trust development associated with positive experiences develops over time and contributes to engagement, satisfaction and treatment adherence (Garrubba and Yap 2019). In this study, determinants of trust are evidenced through communication strategies, nurse knowledge and expertise, promoting safety, accurately responding to individual needs, targeted responses to stress, and supporting decision making (Garrubba and Yap 2019). Feelings of safety were associated with developing trust and the transparency demonstrated through nurse attributes, knowing what to expect, and that the nurse would follow through on what they had promised. Communication is the most significant in the development of trust, with health care professionals also central to patient‐centered care, which is associated with improved outcomes and perceptions of care quality (Kwame and Petrucka 2021; Birkhäuer et al. 2017). Trust was associated with communication that was clear, succinct, consistent, validated, demonstrated empathy, kindness, and active listening in this study. Study findings suggest that TIC is an intervention aligned with developing trust and rapport in health service delivery.

Evidence from this study highlights the benefits of empowerment in overcoming power imbalances, responding to individual safety needs, and advocacy to minimise re‐traumatisation by protecting the patient from unnecessarily retelling their story consistent with the evidence for overcoming service barriers (De Boer et al. 2022). In this study, empowerment through nurse advocacy involved articulating patient needs without disclosing sensitive information. This TIC strategy enables overcoming known barriers to disclosing and accessing health services (Sable et al. 2006).

The importance of service navigation and facilitating access identified in this study is consistent with De Boer et al.'s (2022) findings that key support involves helping gather information to address areas of difficulty and meet a range of needs, illustrating collaboration from the SAMSHA framework (2014); and is pivotal to patient recovery. Improved access and experiences associated with transforming emotional distress and overwhelming feelings into positive experiences through TIC, as identified in this study, provide valuable insights for implementing TIC in health not previously described. Implementation of TIC aligns with safety strategies to address occupational violence in health care settings, offering additional benefits for the sector (Beattie et al. 2019). Such principles can be adopted in other health care settings by activating health care workers with TIC training to demonstrate understanding, build trust, provide individualised responses, and strengthen their role as advocates to make health care accessible.

Findings from this study provide valuable evidence about trauma survivor experiences of TIC in a community health setting. Given that 75% of Australians experience a traumatic event in their lifetime (Productivity Commission 2020), and the evidence to support TIC for improving health engagement, experiences and removing barriers to health access, several recommendations are derived from this study.

### 5.3 Advancing TIC in Health

Currently, TIC is available to those accessing specialist services or living with social inequities; these data have potential policy implications for adopting TIC as a standard across all health services, as anyone receiving health care should expect to feel safe. Policy should specify that trauma‐exposed individuals' symptoms are recognised, and individuals receive adequate support when seeking physical health care; this aligns with trauma care policy recommendations for integrating mental and physical health care (Kleber 2019). While TIC in professional degree programmes is increasing, a recommendation from the findings of this study is that all health professionals should be able to function within this framework (Parker and Johnson‐Lawrence 2022). Additionally, it is recommended that TIC be incorporated into the education and training for new and existing health care workers, as a powerful method of activation in TIC, to ensure they understand the impact of trauma, have the skills to address it, and educate patients (Schimmels and Cunningham's 2021). Opportunities for future research include further exploring the potential for TIC to build patient trust in health care workers, exploring the implementation of TIC across health care services from clinicians' perspective and investigating the impact of TIC on longitudinal health and wellbeing outcomes.

### 5.4 Strengths and Limitations

Findings from this study are limited to the experiences of one male and five female English‐speaking, able‐bodied participants over 18 years of age living in Australia. It is unclear how well they would reflect experiences of diverse populations. The cultural, gender and historical elements of TIC were not included in the study prompt, and the peer support was unable to be included due to service design. As a result of the small sample size, there was an increased risk of sampling and researcher bias, although every effort was made to minimise this risk. Due to the small, purposefully recruited participant group, generalisability is substantially limited. The researchers in this study have nursing qualifications and acknowledge their positionality as white, educated, middle‐class women, which may have influenced the analysis of findings. Study findings are specific to people with lived experience of family violence and sexual assault and did not include known exposure to other traumatic experiences, but findings are consistent with the literature across broader experiences of trauma. Despite this, there is a need for future research with a larger, more robust sample to gain a comprehensive understanding of how to operationalise TIC in healthcare settings. Whilst the study findings are limited to patient experiences of TIC, to the authors' knowledge, there appears to be no evidence of negative impacts from implementing TIC, and there are potential benefits for all patients of healthcare services (Oral et al. 2020).

## 6 Conclusion

This study demonstrated that in patients with exposure to sexual assault or family and domestic violence trauma, the implementation of TIC was associated with enhanced service engagement, positive experiences of care and overcoming barriers to access. TIC supported patients feeling safe and heard through an understanding of trauma, individualised care, trust and the use of advocacy to increase health care accessibility. While experiences of trauma are prevalent within Australia, everyone accessing health care should expect to feel safe and adequately supported when receiving care. Recommendations from this study include policy considerations for trauma support across mainstream health services, the incorporation of TIC as standard care in health services, and education of health professionals to promote the routine application of the TIC framework.

## Author Contributions

V.N.R.: conceptualisation, data curation, formal analysis, investigation, methodology, visualisation, writing (original draft), writing (review and editing). A.M.H.: conceptualisation, formal analysis, investigation, methodology, visualisation, writing (review and editing). R.L.F.: conceptualisation, formal analysis, investigation, methodology, visualisation, writing (review and editing).

## Ethics Statement

This study was registered with the Human Research Ethics Committee [Identifier HREC/77451/MonH‐2021‐281029(v2)].

## Conflicts of Interest

The authors declare no conflicts of interest.

## Supporting information


**Data S1:** nop270331‐sup‐0001‐Supinfo01.docx.

**Data S2:** nop270331‐sup‐0002‐Supinfo02.docx.

## Data Availability

The data that support the findings of this study are available on request from the corresponding author. The data are not publicly available due to privacy or ethical restrictions.
