# Supplementary material for: The Impact of Trauma‐Informed Care on Patient Engagement, Experience and Barriers to Care: A Qualitative Study: Empirical Research Qualitative
Source: Nurs Open. 2025 Sep 25;12(9):e70331. doi: 10.1002/nop2.70331 (PMC12461756; doi:10.1002/nop2.70331)
Supplement: Supplementary file 1 — Data S1: nop270331‐sup‐0001‐Supinfo01.docx. [file NOP2-12-e70331-s002.docx]

**Introduction**

Thank you for taking the time to speak to me today about your experience of the Community Health nursing service supporting people who have an experience of sexual assault or family violence. I just wanted to tell you a couple of things about the project before we get started with the interview.  The first thing is that I want to reassure you that although we will be discussing your experience with the community health service, I won’t be asking you about any specific details about your health conditions or experience you have had related to family violence or sexual assault.  I will simply ask you some questions about seeking health support.

You may see me write down the occasional note while you are speaking, I am simply doing that to remind me to ask you more about something you have said. At the end of the interview, I will need get you to fill in a couple of details about yourself on a survey that won’t have your name and I need to make sure  we have a current address for you so that I can mail you your eGift card.

May I confirm that you've read the Participant Information Sheet? Do you have any questions about the interview or the research?

I'd like to remind you that you may cease the interview at any point and/or withdraw from the study at any time, without any repercussions. You may also decline to answer any questions you'd prefer not to answer.

Are you happy for the interview to be audio-recorded? (Commence audio-recording)

For the recording, could you please confirm that you consent to participate in the interview?

You will have seen from the Plain Language Statement that your participation in this interview is voluntary, and that if you want to pause or stop the interview at any time, please just let me know.

How does a trauma informed care approach to care increase engagement, reduce barriers and enhance overall client experience?

How did you find out about the community health nurse service?

What motivated you to receive support from the MDC community health nurse?

What made it easy for you to work with the MDC nurse?

What other health services have you accessed prior to the MDC nursing service?

Can you tell us about anything that may have made accessing health services difficult before using the MDC nursing service?

Can you describe your experience with the MDC nursing service?

How would you compare the MDC nursing service to other experiences of health services?

Is there something that the MDC nursing service provided that is different to your other experiences of health?

When working with the MDC nurse, what have you found valuable?

When working with the MDC nurse, what did you not find valuable?

What could be done differently?

Is there anything else you would like to tell me about the MDC nursing service?
