# Supplementary material for: The Impact of Trauma‐Informed Care on Patient Engagement, Experience and Barriers to Care: A Qualitative Study: Empirical Research Qualitative
Source: Nurs Open. 2025 Sep 25;12(9):e70331. doi: 10.1002/nop2.70331 (PMC12461756; doi:10.1002/nop2.70331)
Supplement: Supplementary file 2 — Data S2: nop270331‐sup‐0002‐Supinfo02.docx. [file NOP2-12-e70331-s001.docx]

Exploration of trauma informed care by community health nurses supporting victim/survivors of sexual assault and/or family violence

# Distress and Disclosure Protocol - interview

**This protocol is to be used if participants are distressed at any stage during the interview.**

If a participant should become distressed during an interview, the researcher will inform the participant that they can leave, pause or stop the interview. The researcher will use her professional skills to provide immediate support and attempt to alleviate any distress.

The following steps should be followed:

Listen to the participant and allow them to determine the reason for their distress. Grant an opportunity for the participant to express their feelings as they feel comfortable to do so, and attend to practical matters eg. tissues, leaving the space etc., assisting her/him to gain a sense of control over self and environment, assisting access to and planning for support and follow-up eg. support resources.

The researcher and the participant should discuss the appropriateness of continued participation in an interview, or to opt out of the project. The focus at all times should be on supporting the participant to place their own needs before the interest of the project.

If the participant did want to continue with the project, the researcher will consult with supervisors Alison Hutchinson and Renee Fiolet to determine an appropriate course of action to ensure safety for the participant. This would be conveyed to the participant before any action is taken by the researcher.

The participant would be reminded of the following counselling and advocacy services.

# Sexual Assault Crisis Line Ph. 1800 806 292

**Domestic Violence Crisis Line Ph. 1800 015 188**

**Lifeline Ph. 13 11 14**

Any adverse events will be reported to the supervisory panel and ethics committee for review of protocols and procedures and to implement changes as required.
